# Deciphering the Mechanism of Continuous Sacral Erector Spinae Block: A Cadaveric Study

**DOI:** 10.5152/TJAR.2022.21294

**Published:** 2022-12-01

**Authors:** Sandeep Diwan, Rajendra Garud, Parag Sancheti

**Affiliations:** 1 Department of Anaesthesia, Critical Care and Pain, Sancheti Institute for Orthopaedics and Rehabilitation, Pune, India; 2 Department of Anatomy, Bharati Vidyapeeth University Medical College, Pune, India; 3 Department of Knee and Sports Medicine, Sancheti Institute for Orthopedics and Rehabilitation, Pune, India

To the editor,

The sacral erector spinae block (S-ESPB) is a recently described technique, used mostly for analgesia of the posterior perianal area.^1,2^ Albeit the similarity in the name, sacral erector spinae plane (ESP) is anatomically different from the thoracic ESP. The thoracic ESP is bounded anteriorly by the intertransverse ligaments, costotransverse ligaments, and the short rotator muscles which prevent ventral spread of local anaesthetic (LA). The sacral ESP on the other hand is bound anteriorly by the sacral plate which contains the sacral foramina which might be a potential conduit between the dorsal and the ventral aspects of the sacrum. It has recently been termed as a sacral multifidus block (SMFB) as the sacral multifidus (SMF) is the only erector spinae group muscle present in the sacral area unlike the thoracic or lumbar ESP.^3^ The mechanism of SMFB is still unclear. Through cadaveric dissections we attempted to understand the spread of injectate in SMFB.

The study was conducted in the Anatomy Department of a Medical College in India. In 3 formalin-preserved cadavers, continuous bilateral SMFB were performed. With the cadaver in prone position, a curvilinear ultrasound transducer was placed in a midline longitudinal orientation to identify the fifth lumbar (L5) spinous process and sacrum. The transducer was shifted laterally and caudally to identify the second sacral foramen (SF-2), which was visualized as a “break” in the continuity of the sacral outline.^4^ An 18G Tuohy needle was inserted in a cranial-to-caudal direction and the needle tip was positioned deep to the SMF muscle above the SF-2. Three millilitres of 0.9% saline was injected to confirm the needle location. Subsequently, a solution of 0.5 mL methylene blue dye (MBD) diluted in 10 mL of 0.9% saline was injected and the catheter was inserted to a distance of 3 cm. A further 10 mL solution was injected through the catheter and the catheter was removed 1 hour later. With all the cadavers positioned prone, cadaver 1 was dissected on its dorsal aspect in the sacral region, cadaver 2 underwent axial sections at the level of sacroiliac joints at 10 mm intervals, and cadaver 3 was sectioned in a sagittal plane through the sacral epidural space (Table 1). Open dissection revealed MBD in the lumbar multifidus muscle, the SMF and beneath it. Dissection beneath the SMF revealed that the dye reached as far as the dorsal sacral foramina. Axial section revealed MBD above and beneath the SMF muscle and around the sacral nerve roots. The sagittal section depicted the path of the dye as it surrounded the SMF muscle and reached the sacral canal staining the sacral nerve roots (Figure 1). It appeared that the MBD percolated through the mesh of muscle and ligaments over the dorsal surface of the sacral foramina into the sacral epidural space. A continuous infusion built up volume-related pressure in the compact and less compliant SMF plane leading to a diffusion of the injectate into the epidural space and more ventrally into the sacral plexus. The collapsed SMF plane, devoid of water content in the connective tissue, facilitated translocation of MBD from dorsal to ventral areas. However not all the sacral nerve roots were stained and it was the third and fourth sacral nerves in both the cadavers. We deduced that the distribution depends on the site of the needle tip and the catheter tip at the time of injection in relation to the dorsal sacral foramina.

Isolated case reports^5-7^ and series have demonstrated that the SMFB could be successfully implemented as a part of postoperative multimodal analgesia. Needle position for S-ESPB has been described in 3 areas- medial and inferior at the second sacral vertebra (S2), medial and superior at the third sacral vertebra (S3), and in the midline at the level of the fourth sacral vertebra (S4) for chronic perianal pain. Without a supporting radiological or a cadaveric study, the authors suggested an epidural diffusion and ventral spread to block the anterior rami as the mechanism of action of SMFB.

Cadaveric injections in our study demonstrated a consistent spread to the SMF plane along with some inconsistent spread in sacral epidural space and along the intra-pelvic ventral nerve roots. Thus, an SMFB is predominantly a dorsal sacral rami block. The inconsistent and uncertain spread in the sacral epidural space and along the sacral nerve roots possibly explain its mechanism of action for perineal surgical procedures. The lack of space in the SMF plane and the potential passage through the sacral foramina indicates that the Insertion of catheter and higher injection pressure may facilitate ventral spread of the injectate.

There were several limitations. The number of cadavers used was less. We understand the limitations of formalin preserved cadavers. Studies performed on fresh cadavers can be more precise. Moreover, there was no radiological imaging to investigate the extent and path of spread. Although we used 20 mL volume here, we are still not sure about the optimal volume for this block. Further studies are recommended in fresh cadavers or Theil embalmed cadavers along with clinical imaging study with radiocontrast to establish the mechanism of action and optimal volume of SMFB.

## Figures and Tables

**Figure 1.**
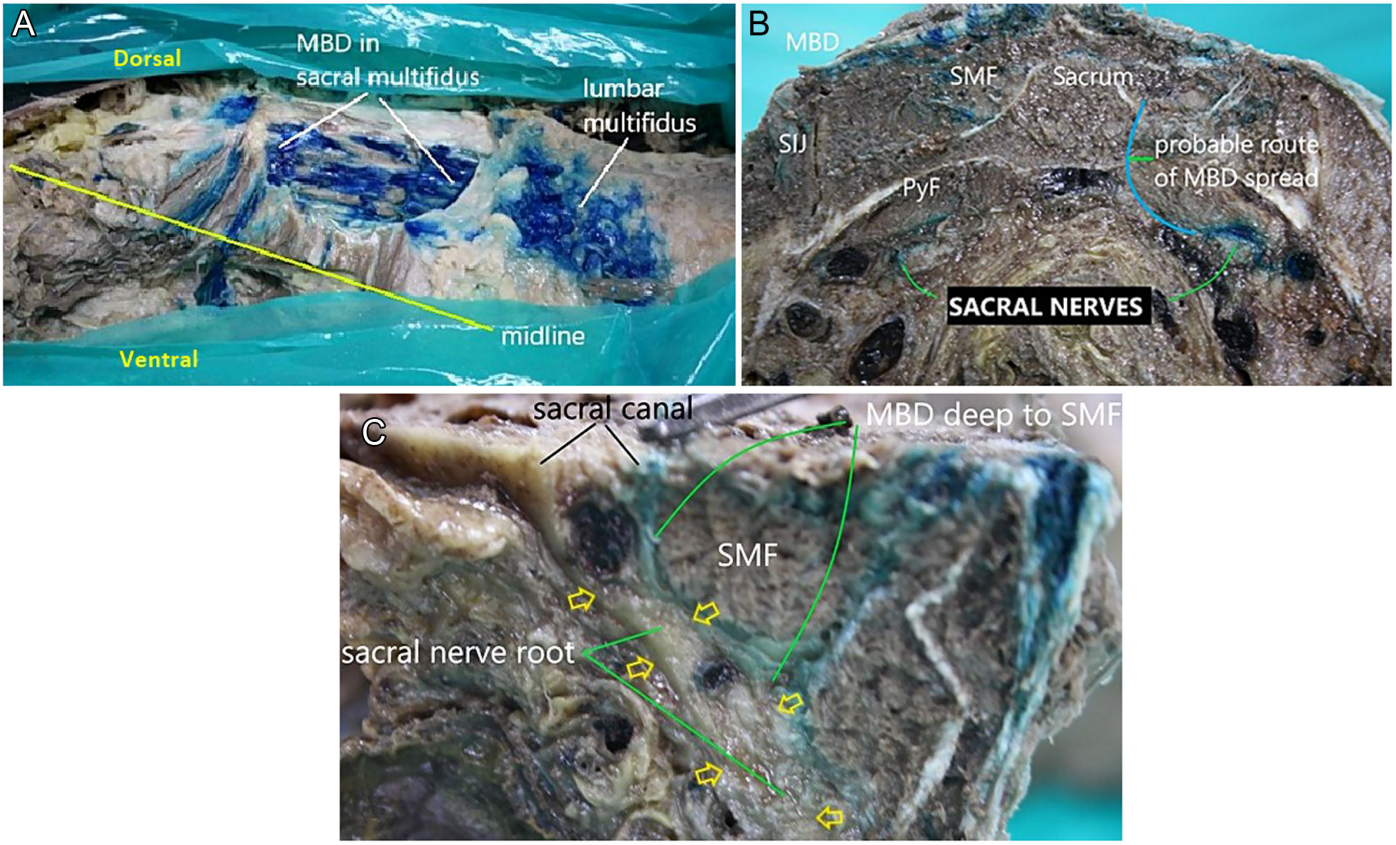
(A) Open dissection of cadaver 1 over the dorsum of sacral bone showing methylene blue dye above and beneath sacral multifidus muscle. (B) Axial section of cadaver 2 at the sacroiliac joint depicting methylene blue dye beneath the sacral multifidus muscle bilaterally, and staining the ventral sacral nerve roots. (C) Sagittal section of cadaver 3 through the sacral epidural space illustrating methylene blue dye engulfing the sacral multifidus muscle, diffusing into the sacral epidural canal and highlighting on the dorsal aspect of the sacral nerve root in the epidural canal. Yellow arrow heads represent the outlines of the sacral epidural space. MBD, methylene blue dye; SIJ, sacroiliac joint; SMF, sacral multifidus muscle; PyF, pyriformis.

**Table 1.** Summary of cadaveric section finding

Cadaver no.	Section	Findings
1	Open dissection	MBD* in the lumbar multifidus, over the sacral multifidus and beneath it as far as the second sacral dorsal foramina
2	Axial section	MBD dorsal and ventral to the sacral multifidus, encircling the sacral nerve roots bilaterally
3	Sagittal section	Diffusion of MBD around the sacral multifidus and spread to the sacral epidural canal and the ventral nerve roots

*MBD: methylene bluedye.
